# Direct Measurement of the Topological Charge in Elliptical Beams Using Diffraction by a Triangular Aperture

**DOI:** 10.1038/s41598-018-24928-5

**Published:** 2018-04-23

**Authors:** Leandro A. Melo, Alcenísio J. Jesus-Silva, Sabino Chávez-Cerda, Paulo H. Souto Ribeiro, Willamys C. Soares

**Affiliations:** 1 Instituto de Física, Universidade Federal de Alagoas, Maceió, Alagoas 57361-970 Brazil; 2 Instituto Nacional de Astrofísica, Óptica y Electrónica, Luis Enrique Erro No.1, Tonantzintla, Puebla, 72840 Mexico; 3 Centro de Investigaciones en Optica, Loma del Bosque 115, León, Gto. 37150 Mexico; 4 Grupo de Física da Matéria Condensada, Núcleo de Ciências Exatas – NCEX, Campus Arapiraca, Universidade Federal de Alagoas, Arapiraca, Alagoas 57309-005 Brazil; 5 Grupo de Informação Quântica do Sul, GIQSul, Departamento de Física, Universidade Federal de Santa Catarina, Florianópolis, Santa Catarina, 88040-900 Brazil

## Abstract

We introduce a simple method to characterize the topological charge associated with the orbital angular momentum of a m-order elliptic light beam. This method consists in the observation of the far field pattern of the beam carrying orbital angular momentum, diffracted from a triangular aperture. We show numerically and experimentally, for Mathieu, Ince–Gaussian, and vortex Hermite–Gaussian beams, that only isosceles triangular apertures allow us to determine in a precise and direct way, the magnitude m of the order and the number and sign of unitary topological charges of isolated vortices inside the core of these beams.

## Introduction

Light beams possessing orbital angular momentum (OAM) have been extensively studied since its first demonstration in 1992^1,2^. Laguerre-Gauss^3^ and Bessel beams^4^ are examples of beams carrying OAM. They can be decomposed in terms of orthogonal components, and it is possible to construct a geometric representation equivalent to the Poincaré sphere for the polarization^5^. These beams have found applications in optical tweezers^6^, singular optical lattice generation^7^, atom traps^8^, transfer of OAM to microparticles^9^, nanostructures and atoms^10^, and for shaping Bose–Einstein condensates^11^. Another important application is the preparation of photons entangled in their orbital angular momentum (OAM) degree of freedom^2,12^, which are candidates for implementing high performance quantum communication^13^.

Elliptical vortex beams (EVBs) have also received considerable attention in recent years^14–18^. This type of beam has an elliptical symmetry which is stable on propagation and it is also promising for all previous applications of circular OAM beams, for instance, the EVBs have been applied in optical trapping and manipulation of particles^19,20^, quantum information^21,22^, and beam shaping in nonlinear media^23,24^. Several EVBs were investigated earlier, including Mathieu^14,15^, helical Ince–Gaussian (HIG)^16^, vortex Hermite–Gaussian (VHG) beams^17^, and elliptic perfect optical vortices^18^. Other works have investigated simple ways of producing EVBs^25,26^. However, the diffraction of these beams by apertures has not been extensively investigated, except a method for measuring the orbital angular momentum of elliptical vortex beams by using a slit hexagon aperture^27^.

We contribute to this type of study, by showing that the order m of an EVB can be determined by inspection of the diffraction pattern from an isosceles triangular aperture. It is known that the topological charge (TC) of circular beams can be determined by interferometric^28–30^ and diffractive^31–37^ methods. For Laguerre-Gaussian and Bessel beams, the sign and magnitude of the topological charge can be determined by diffraction through an equilateral triangular aperture^38^. We extend this method for EVBs by changing from an equilateral to an isosceles triangular aperture.

We demonstrate that the order m and the beam wavefront helicity sign can be obtained from the diffraction pattern in an unambiguous and direct way up to m = 10. The procedure is only reliable for isosceles triangular apertures. We discuss a practical method to design the most appropriated triangular aperture for this task.

## Results

The theoretical approach for this diffraction problem consists in calculating the far field pattern by a triangular aperture. In order to do that, we use the Fraunhofer integral given by^39^

1 $$E(x,y,z)={E}_{0}\frac{i{e}^{ikz}}{\lambda z}{e}^{i\frac{k}{2z}({x}^{2}+{y}^{2})}{\int }_{-\infty }^{\infty }{\int }_{-\infty }^{\infty }E(x^{\prime} ,y^{\prime} ,0){e}^{i\frac{k}{z}(xx^{\prime} +yy^{\prime} )}dx^{\prime} dy^{\prime} ,$$

where *E(x, y, z)* gives the electric field amplitude at the transverse position with coordinates (*x, y)* in the plane situated at a distance *z* from the diffraction screen. λ is the wavelength in the vacuum, *k* is the wavevector and *E*_0_ is a constant.

As we are interested in the transverse intensity distributions at a fixed plane placed at the position z = z_0_, far enough from the aperture, we can use the scale transformations *K*_*x*_ = *k*.*x*/*z*_0_, *K*_*y*_ = *k*.*y*/*z*_0_, and omit the term outside the integral. Thus, the Fraunhofer integral becomes a Fourier transform,

2 $$E({K}_{x},{K}_{y})={\int }_{-\infty }^{\infty }{\int }_{-\infty }^{\infty }E(x^{\prime} ,y^{\prime} ,0){e}^{-i({K}_{x}x^{\prime} +{K}_{y}y^{\prime} )}dx^{\prime} dy^{\prime} ,$$

where *E(x*′, *y*′, *0)* is the product between the incident field and the aperture function. Due to the elliptical symmetry of the EVBs, the appropriated aperture must have an isosceles triangular shape and it should be placed in the beam as described in more detail below. The longer axis of the aperture should lie along the longer axis of the beam. For integer and circular OAM beams, the integral in Eq. (2) for the triangular aperture can be analytically evaluated^40^. However, for EVBs, analytical solutions were not yet derived. Therefore, we will solve these integrals numerically.

**Figure 1:**
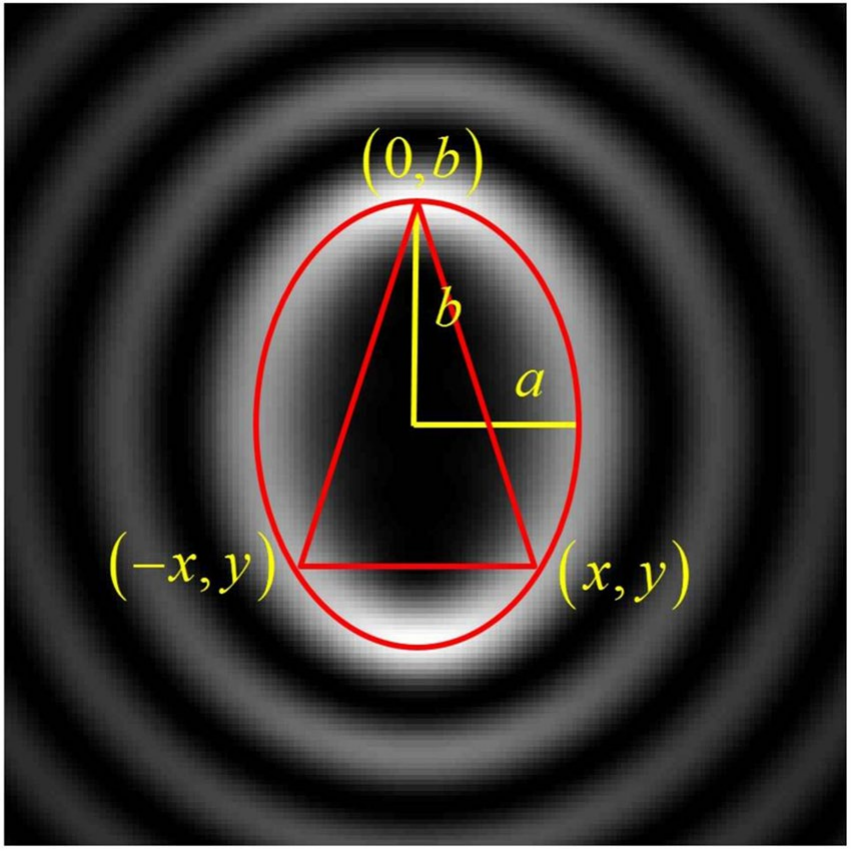
Geometry of the edges of an isosceles triangular aperture inscribed in an ellipse matching the global maxima of the intensity pattern of a Mathieu beam with *m* = 5 and *q* = 6.

In Fig. 1 we show in red, an isosceles triangle representing the aperture inscribed in an ellipse representing the shape of the beam. In order to design the optimal triangle, we need to measure the intensity transverse profile of the beam at the position where the aperture will be placed. From the intensity pattern, we obtain the dimensions of the semi-minor axis *a*_1_ and semi-major axis *b*_1_, which are the distances from the center to the intensity global maxima in the *x* and *y* directions, respectively. This provides us with the equation ${x}^{2}/{a}_{1}^{2}+{y}^{2}/{b}_{1}^{2}=1$ for the ellipse shown in Fig. 1, with eccentricity $e=\sqrt{{b}_{1}^{2}-{a}_{1}^{2}}/{b}_{1}$. In order to obtain the coordinates (−x, y) and (x, y) for the vertices of the triangle, we apply the transformation ${a}_{1}^{^{\prime} }={a}_{1}(1-0.3e)$ and ${b}_{1}^{^{\prime} }={b}_{1}(1+0.3e)$ to obtain $y=-\,{b}_{1}^{^{\prime} }/2$ and $x={a}_{1}^{^{\prime} }\sqrt{3}/2$. This procedure was developed in order to maximize the visibility of the diffraction features that contain the information about the topological charge, as a function of the relative sizes of beam and aperture. When the elliptical beam tends to a circular one, the optimal aperture tends to the equilateral triangle.

In this work, we approach three types of elliptical beams. The helical Mathieu beams are solutions of the Helmholtz equation in the elliptic cylindrical coordinates and can be constructed from a linear combination of even and odd Mathieu functions as^14^

3 $$E(\xi ,\eta ,0)={C}_{m}{\rm{J}}{e}_{m}(\xi ,q)c{e}_{m}(\eta ,q)\pm i{S}_{m}{\rm{J}}{o}_{m}\,(\xi ,q)s{e}_{m}(\eta ,q),$$

where *ξ* and *η* are the radial and angular variables in the elliptical coordinates, J*e*_*m*_(·) and J*o*_*m*_(·) represent the *m*th-order even and odd radial Mathieu functions and *ce*_*m*_(·), *se*_*m*_(·) correspond to the *m*th-order even and odd angular Mathieu functions. *C*_*m*_ and *S*_*m*_ are the normalization coefficients and *q* is a parameter that characterizes the ellipticity of the beam. The sign in Eq. (3) defines the rotating direction of the wavefront.

The HIG modes are solutions of the paraxial wave equation (PWE), also in the elliptic cylindrical coordinates, and can be expressed as a superposition of even and odd Ince–Gaussian modes (IGMs)^16^,

4 $$E(\xi ,\eta ,\varepsilon )=I{G}_{p,m}^{e}(\xi ,\eta ,\varepsilon )\pm iI{G}_{p,m}^{o}(\xi ,\eta ,\varepsilon ),$$

where *ξ* and *η* are the radial and the angular elliptic variables, respectively, and *ε* is the ellipticity parameter. The parameter *p* is related to the number of rings which is given by the relation $1+(p-m)/2$, and *m* gives the overall topological charge. $I{G}_{p,m}^{e}(\,\cdot \,)$ and $I{G}_{p.m}^{o}(\,\cdot \,)$ are even and odd IGMs, respectively.

The VHG beams are formed from a superposition of *n* + 1 generalized Hermite–Gaussian beams^41^, which are solutions of the PWE in Cartesian coordinates, and their complex amplitudes are given by^17^,

5 $$E(x,y)={i}^{m}\exp (-\frac{{x}^{2}}{{w}_{x}^{2}}-\frac{{y}^{2}}{{w}_{y}^{2}}){(\frac{1-{a}^{2}}{1+{a}^{2}})}^{m/2}{H}_{m}[\sqrt{2}\frac{ia{w}_{y}x+{w}_{x}y}{{w}_{x}{w}_{y}\sqrt{1-{a}^{2}}}],$$

where *x* and *y* are the Cartesian coordinates, *m* is the order of the beam, *a* is a real constant that controls the beam ellipticity, *H*_*n*_(·) is the Hermite polynomial, and *w*_*x*_ and *w*_*y*_ are the Gaussian beam waist radii.

**Figure 2:**
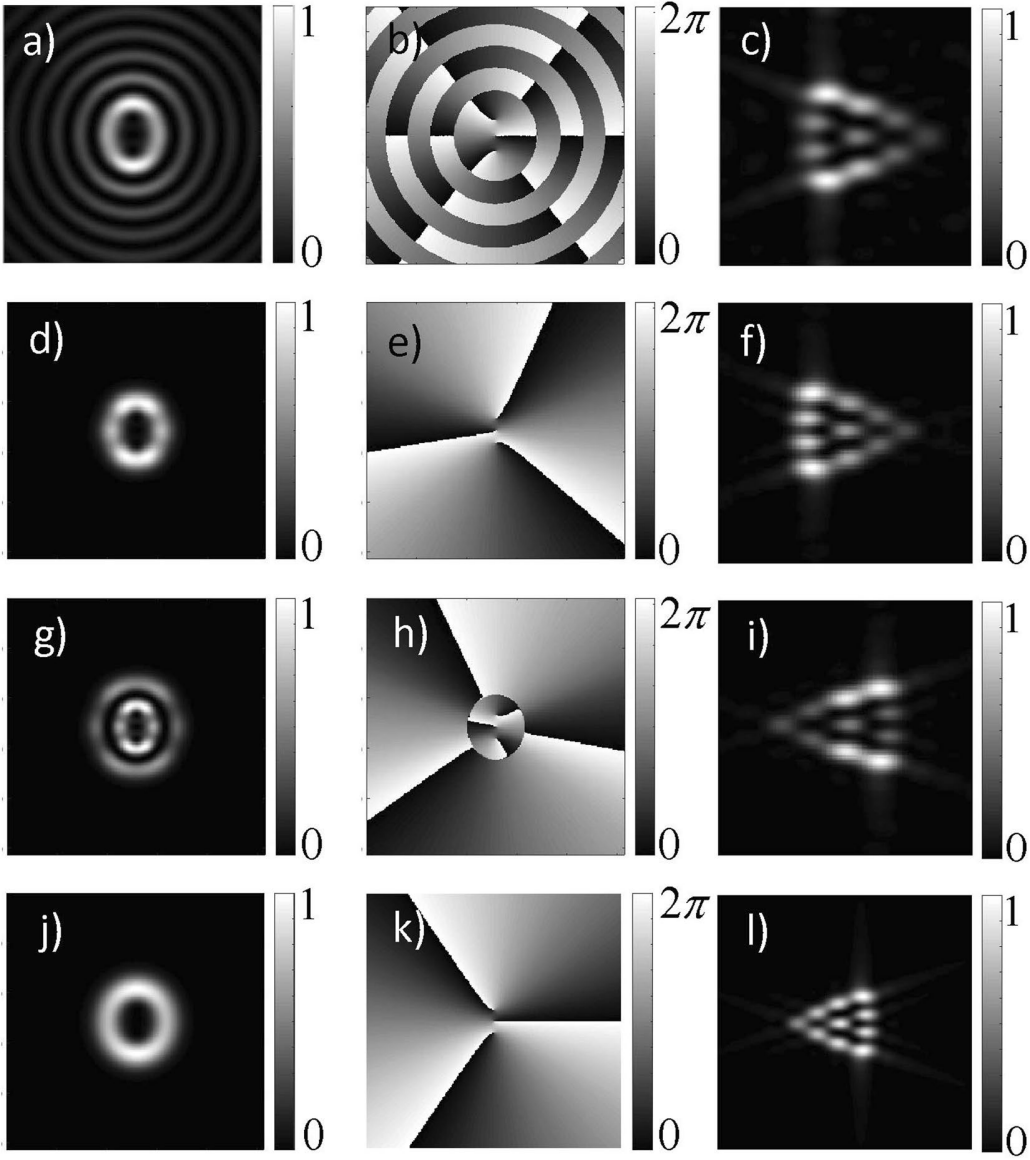
Calculated intensities (left column), phase (center column), and Fraunhofer diffraction patterns (right column) for EVBs. From the top to the bottom, the first row: Mathieu beam with *m* = 3 and *q* = 2; second row: HIG beam with *m* = 3, *p* = 3 and *ε* = 1; third row: HIG beam with *m* = 3, *p* = 5 and *ε* = 1; fourth row: VHG beam with *m* = 3 and *a* = 0.80. From second to third rows we see the effect of changing the sign in Eq. (4).

Figure 2 shows the theoretical transverse intensity (left column), phase (center column), and Fraunhofer diffraction patterns (right column) for EVBs. Figure 2(a)–(c) correspond to a Mathieu beam with *m* = 3 and *q* = 2, Fig. 2(d)–(f) correspond to a HIG beam with *m* = 3, *p* = 3 and *ε* = 1, Fig. 2(g)–(i) correspond to a HIG beam with *m* = 3, *p* = 5 and *ε* = 1, and Fig. 2(j)–(l) correspond to a VHG beam with *m* = 3 and *a* = 0.80. For all of these modes, the sign of *m* does not determine the helicity, or the sense of rotation of the wavefront. For Mathieu and HIG beams the helicity depends on the sign of the imaginary term in Eqs (3) and (4), while for VHG beams it depends if the parameter *a* in Eq. (5) is bigger or smaller than 1. In Fig. 2(e) and (h), the effect of changing the sign in Eq. (4) is illustrated. In Fig. 2(e) the sense of increasing phase is clockwise, while in Fig. 2(h) it is counterclockwise. The phase distribution maps in Fig. 2(b),(e),(h) and (k), also illustrate the fact that the *m*th-order EVB with nonzero eccentricity contains *m* in-line vortices, each one with unitary topological charge of the same sign such that the modulus of the total charge is *m*.

The patterns 2(c), 2(f), 2(i), and 2(l), resulting from diffraction through an isosceles triangular aperture allow us to determine *m* and the sign of the unitary vortices at the beam core. The number of bright spots is directly related to *m*, and the sign is given by the orientation of the pattern. According to the simulations, a safe region for the method to work is in the range of 0 < *e* ≤ 0.8 and *m* ≤ 10. Out of these limits the patterns are not truncated and we cannot properly count the number of spots anymore.

**Figure 3:**
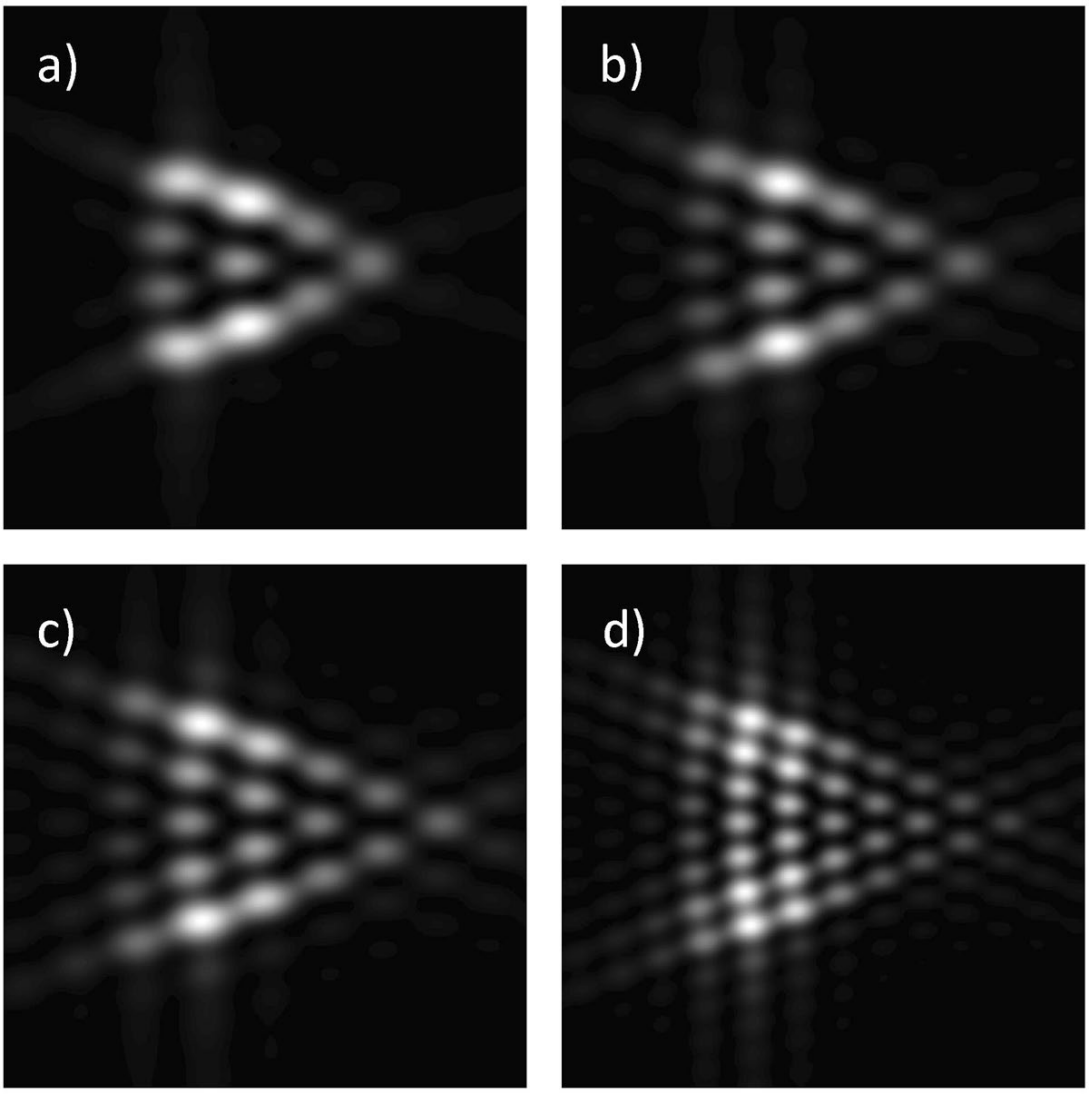
Fraunhofer patterns of Mathieu beams diffracted by a triangular aperture. (**a**) *m* = *3, q* = *2*, (**b**) *m* = *4, q* = *3*, (**c**) *m* = *5, q* = *4*, and (**d**) *m* = *7, q* = *7*. Notice that each side has *m* + *1* bright spots.

In Fig. 3 we show the numerically computed diffraction patterns for the *mth*-order input Mathieu beams. Comparing the diffraction patterns for different values of *m*, it is possible to establish a rule to determine the order of the beams, in the same way as for the HIG and VHG beams. We can observe that the value of *m* is directly related to the first order external diffraction lobes (maxima) formed on the sides of the triangle. The total charge is given by *m* = *N* - 1, where *N* is the number of lobes on anyone of the sides of the triangle. This is valid for all EVBs studied here.

**Figure 4:**
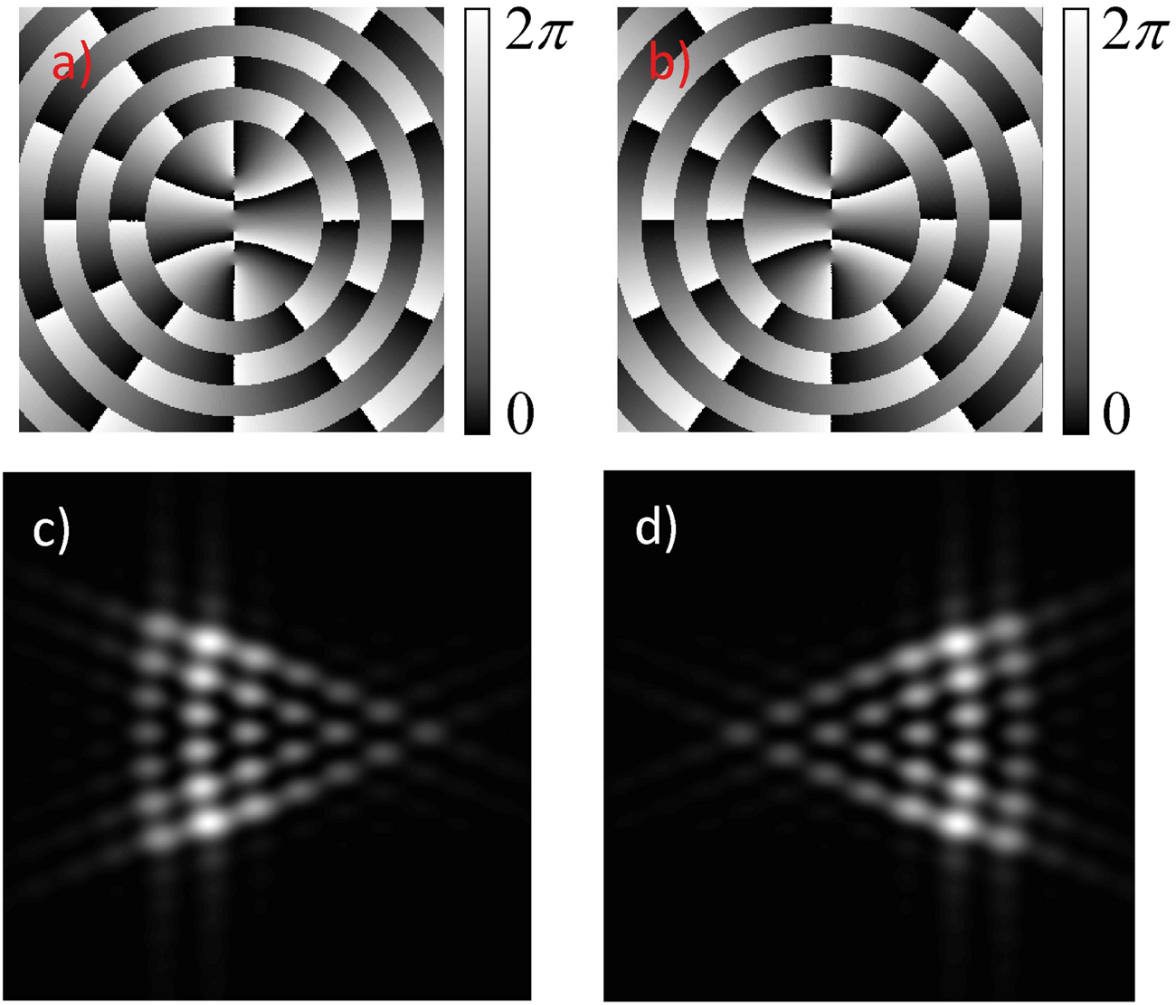
Effect of changing the sign in Eq. 3 in the Fraunhofer diffraction patterns. (**a**) and (**c**) *m* = *6*, *q* = *6* for plus sign (clockwise increasing phase); (**b**) and (**d**) *m* = *6*, *q* = *6* for minus sign (counterclockwise increasing phase).

Figure 4 illustrates the effect of changing the sign of the imaginary part in Eq. (3) for a Mathieu beam, with clockwise rotation (plus sign in Eq. (3)), in Fig. 4(a) and (c), and counterclockwise rotation (minus sign in Eq. (3)), in Fig. 4(b) and (d).

So far, we have shown numerically that the diffraction pattern through an isosceles triangular aperture determines the total topological charge *m*, and the helicity of Mathieu, HIG and VHG beams in a clear and unambiguous way. In order to obtain this result, we have analyzed other geometries for the diffraction aperture like a lozenge for instance, but our studies demonstrated that the isosceles triangle is the most appropriated geometry. This is similar to what happens for circular beams, for which other geometries like a square aperture, for instance, can determine the modulus of the topological charge but not the sign^25^. In this last case, only the equilateral triangle can provide the complete information about *m* and its sign^38^.

**Figure 5:**
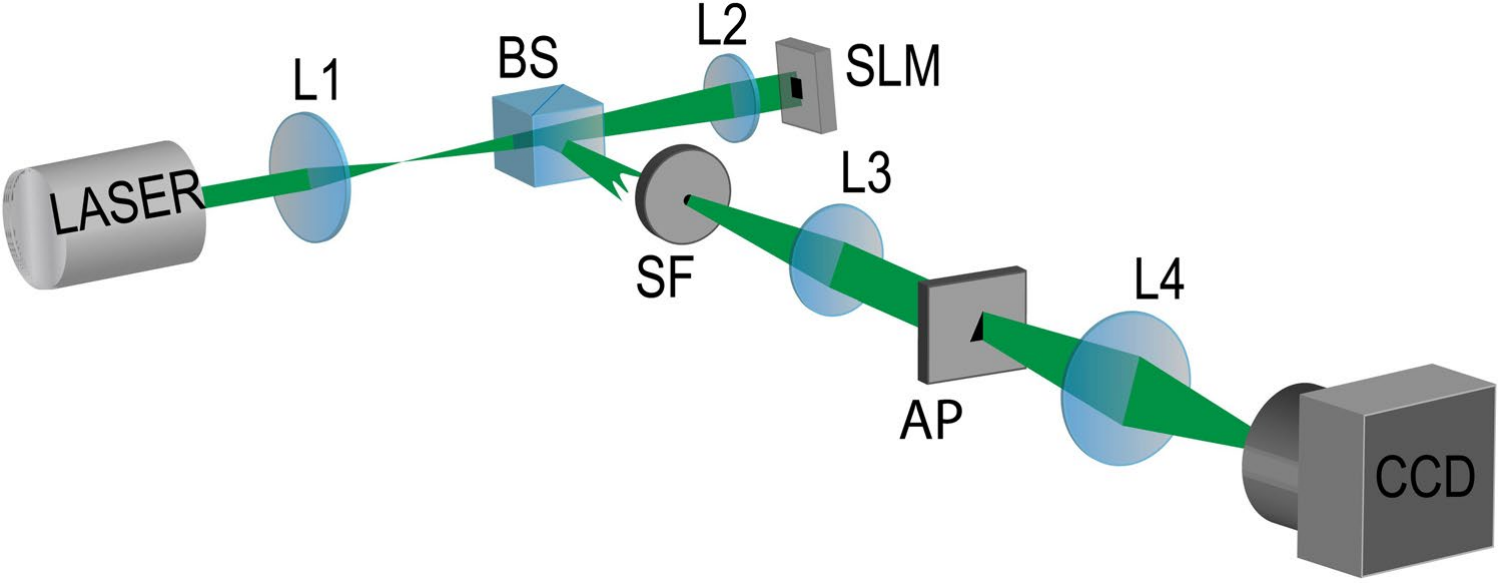
Experimental Setup: *L*1, *L*2, *L*3 and *L*4 are lenses, BS is a 50/50 beam splitter, SLM is a spatial light modulator, SF is a spatial filter, AP is the triangular aperture and CCD is a camera.

We have performed an experiment, in order to confirm our numerical results. Figure 5 shows the sketch of the experimental setup, which is described in detail in section “Methods”. We have diffracted Mathieu, HIG and VHG beams through an isosceles triangular aperture, and demonstrated the validity of our method to determine the order *m* of EVB beams.

**Figure 6:**
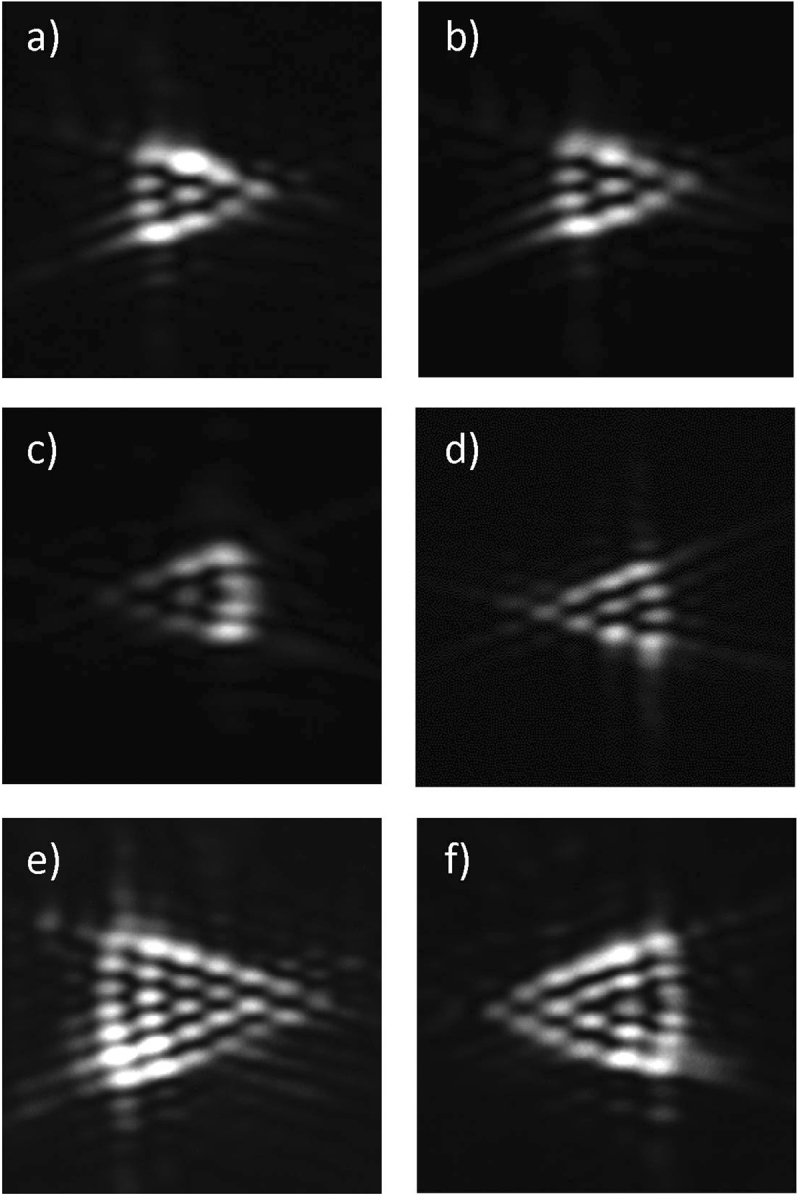
Experimental results for the intensity distribution of the diffraction patterns for EVBs: (**a**) Mathieu beam with *m* = 3 and *q* = 2, (**b**) HIG beam with *m* = 3, *p* = 3 and *ε* = 1, (**c**) HIG beam with *m* = 3, *p* = 5 and *ε* = 1, (**d**) VHG beam with *m* = 3 and *a* = 0.80, (**e**) Mathieu beam with *m* = 6 and *q* = 6, and (**f**) HIG beam with *m* = 5, *p* = 9 and *ε* = 1.

In Fig. 6, we show the experimental results. The triangular structures are the diffraction patterns and each side of the triangles has *m* + *1* bright spots as theoretically predicted. These results confirm our numerical results and demonstrate the use of diffraction patterns by triangular aperture to determine the order of EVBs. They also confirm that the information about the helicity of the wavefront is given by the orientation of the triangular pattern. Different from the traditional circular modes, e.g. Laguerre Gauss and Bessel beams^38^, the sense of wavefront rotation is not determined by the sign of *m*. We have found a very good agreement between theory and experiment.

## Conclusion

In summary, we have numerically and experimentally demonstrated a technique that allows us to determine the order of an EVB in an unambiguous way. We have also presented a recipe to design the optimal triangular aperture for this measurement. This non–interferometric technique requires only simple measurements of intensity patterns. The value of *m* is determined by counting the number of lobes in anyone of the sides of the triangular diffraction pattern. The sense of wavefront rotation can also be determined by the orientation of the diffraction pattern.

## Methods

The experimental setup is shown in Fig. 5. Different orders and types of EVBs are generated from an initial Gaussian mode of an Argon Laser operating at 514 nm. The beam is expanded by a factor of about 17, using lenses *L1*, with focal length *f*_*1*_ = 30 *mm*, and *L*2, with focal length *f*_2_ = 500 *mm*. The expanded beam illuminates a computer-generated hologram^42^ displayed in a spatial light modulator (SLM) (Hamamatsu Model X10468-01). The 50/50 beam splitter (BS) in between *L1* and *L2* is used to allow normal incidence in the SLM. For each type of EVB there is a corresponding type of hologram in the SLM. The reflected beam from the SLM is focused by lens *L*2 in the plane of the spatial filter (SF) after reflection by the BS. The spatial filtering selects the desired diffraction order from the SLM. Lens *L*3, with focal length *f*_*3*_ = *300* *mm*, collimates the beam again, which is incident on the isosceles triangular aperture (AP). It is mounted in a xyz translation stage for precise alignment with respect to the light beam. Finally, lens L4, with focal length *f*_4_ = 200 *mm*, is used to implement the optical Fourier Transform of the field in the aperture plane onto the CCD detection plane. This is the physical realization of the integration in Eq. (2). The transverse intensity patterns corresponding to the Fraunhofer diffraction are registered.
